# Simultaneous Determination of Vitamin D and Its Hydroxylated and Esterified Metabolites by Ultrahigh-Performance Supercritical Fluid Chromatography–Tandem Mass Spectrometry

**DOI:** 10.1021/acs.analchem.1c04016

**Published:** 2022-02-09

**Authors:** Bárbara Socas-Rodríguez, Veronika Pilařová, Margareta Sandahl, Cecilia Holm, Charlotta Turner

**Affiliations:** † Department of Chemistry, Centre for Analysis and Synthesis, Lund University, P.O. Box 124, Lund 22100, Sweden; ‡ Department of Analytical Chemistry, Faculty of Pharmacy in Hradec Králové, Charles University, Akademika Heyrovského 1203, Hradec Králové 500 05, Czech Republic; § Department of Experimental Medical Science, Faculty of Medicine, Lund University, P.O. Box 124, Lund 22100, Sweden

## Abstract

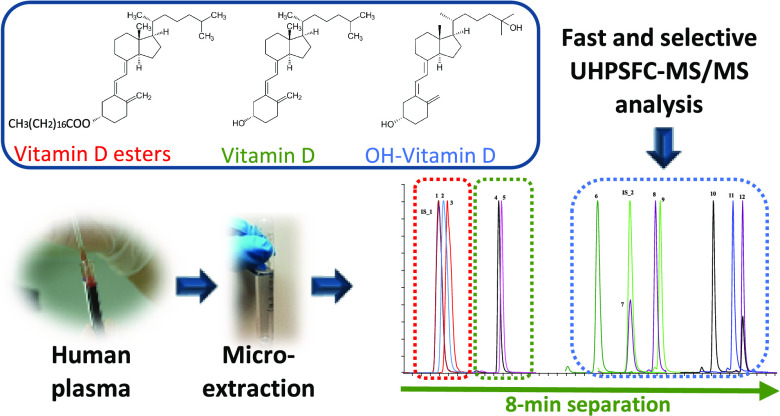

In this study, an analytical method has been developed that, for the first time, allows simultaneous determination of vitamin D_2_ and vitamin D_3_ along with their hydroxylated and esterified forms. A group of 12 vitamin D analogues including vitamin D_2_ and vitamin D_3_, seven hydroxylated metabolites, and three ester forms were separated in a single 8.0 min run using ultrahigh-performance supercritical fluid chromatography coupled with triple quadrupole tandem mass spectrometry. Electrospray ionization and atmospheric pressure chemical ionization were investigated as ion sources, of which the latter showed a higher ionization efficiency. Chromatographic conditions were thoroughly evaluated by a step-by-step method, whereas an experimental design was applied for the optimization of the ionization parameters. Calibration and repeatability studies were carried out to validate the instrumental methodology showing determination coefficients higher than 0.9992 and good intra- and interday precision with relative standard deviations for areas and retention times lower than 10 and 2.1%, respectively, for all target analytes. Limits of quantification were below 3.03 μg/L for all compounds. The methodology was then validated and applied for the evaluation of human plasma samples in order to demonstrate its applicability to the analysis of vitamin D analogues in biological samples. Samples of five individuals were analyzed. Results show that linoleate-D_3_, vitamin D_2_, vitamin D_3_, 25-hydroxyvitamin D_2_, 24,25-dihydroxyvitamin D_3_, and 1,25-dihydroxyvitamin D_3_ could be detected in most samples, while the two latter also were quantified in all analyzed samples.

Vitamin D_3_ (D_3_), vitamin D_2_ (D_2_), and their metabolites constitute a relevant group of fat-soluble vitamins involved in calcium homeostasis, bone metabolism, and other important physiological processes in different tissues and organs of the human body.^1^ Many studies have pointed out the relationship between low vitamin D status, caused by insufficient sun exposure and/or insufficient dietary intake, and the development of bone diseases. In addition, vitamin D insufficiency has been associated with extra-skeletal disorders, such as infectious and autoimmune diseases, autism, cardiovascular disorders, diabetes, and even several types of cancer.^1,2^

It is well known that D_3_ can be synthesized from its precursor (7-dehydrocholesterol) in the skin by the effect of ultraviolet B radiation. It can also be obtained through dietary intake and absorption in the intestine. Vitamin D_3_ and D_2_ are hydroxylated in the liver to form 25-OH-D, which is the target compound used today in the clinic for the estimation of vitamin D status. In the kidney, 25-OH-D is hydroxylated to form 1,25-(OH)_2_-D, which is the biologically active form of vitamin D. There are also other metabolites, including esters, but analytical methods for them are still lacking and their biological role remains elusive.^1,3^ Thus, there is an important gap in knowledge with regard to how and where esters of vitamin D are formed, the enzymes involved in their synthesis and regulation, and the regulation of these enzymes. Vitamin D deficiency and insufficiency are a global health issue.^4^ It has been estimated that more than one billion adults and children worldwide are afflicted by vitamin D deficiency or insufficiency. The prevalence is higher among obese individuals, which has led to formulation of the hypothesis of sequestration of vitamin D in adipose tissue.^5^

In view of the global pandemic of vitamin D deficiency and insufficiency, methods that allow rapid and simultaneous determination of the different vitamin D metabolites in plasma with high sensitivity are urgently needed in the clinic. In addition, methods that allow the determination of different vitamin D metabolites, including esters, in tissues are needed to elucidate how vitamin D is handled in tissues and thereby understand why vitamin D insufficiency is more prevalent among the obese.

In the past years, targeted analysis of these compounds have been done usually by liquid chromatography (LC) combined with conventional detectors and/or mass spectrometry (MS).^6^ All these methods have been focused on the determination of D_3_^7^ or hydroxylated metabolites.^8−13^ However, the analysis of ester forms has not been carried out so far in any type of sample, as described in a recent review article.^6^ The advantages of supercritical fluid chromatography (SFC) in terms of selectivity, comprehensiveness, and analysis speed together with its compatibility with a large range of different detectors have identified this technique as a promising alternative to LC.^14^ In this regard, SFC hyphenated to MS has been applied for the determination of vitamin D analogues in our own studies^15^ and in others^16−18^ but not including both hydroxylated and ester forms. It is clear from the literature that the use of polar functionalized stationary phases in SFC enables the separation of analytes with a great variety of polarities with Log P between −2 and 10.^18^ Hence, there is an obvious potential of this technique to address the simultaneous separation of vitamin D esters and hydroxylated forms in the same run. Additionally, its coupling with MS using a selective and sensitive analyzer such as a triple quadrupole (QqQ) highly enhances the potential of the analytical technique in targeted analysis.

To the best of our knowledge, there is no previous work on the simultaneous separation of vitamin D derivatives including hydroxylated and ester metabolites, and there is not even a method for the analysis of ester forms separately. Consequently, the role of vitamin D esters and some other less frequently studied vitamin D metabolites in diseases associated with vitamin D insufficiency is completely unknown. For this reason, in this work, we propose a new methodology based on the combination of ultrahigh-performance supercritical fluid chromatography-tandem mass spectrometry (UHPSFC-MS/MS) and a miniaturized liquid–liquid extraction (LLE) method for the extraction and determination of a group of 12 vitamin D analogues (i.e., D_2_, D_3_, 1-hydroxyvitamin D_2_ (1-OH-D_2_), 1-hydroxyvitamin D_3_ (1-OH-D_3_), 25-hydroxyvitamin D_2_ (25-OH-D_2_), 25-hydroxyvitamin D_3_ (25-OH-D_3_), 24,25-dihydroxyvitamin D_3_ (24,25-(OH)_2_-D_3_), 1,25-dihydroxyvitamin D_2_ (1,25-(OH)_2_-D_2_), 1,25-dihydroxyvitamin D_3_ (1,25-(OH)_2_-D_3_), palmitate-D_3_, linoleate-D_3_, and stearate-D_3_) in human plasma samples, thus establishing a first effort toward a fast, comprehensive, and selective analysis method to be used in analysis labs in research and hospital settings.

## Experimental Section

### Chemicals and Materials

Analytical standards of D_2_, D_3_, 1-OH-D_2_, and 1-OH-D_3_ were acquired from TCI Europe N.V. (Eschborn, Germany); 25-OH-D_2_, 25-OH-D_3_, and 24,25-(OH)_2_-D_3_ from Sigma-Aldrich Chemie (Steinheim, Germany); 1,25-(OH)_2_-D_2_ from Cayman Chemicals (MI, USA); 1,25-(OH)_2_-D_3_ from Carbosynth (Berkshire, UK); and 25-OH-D_3_-^13^C_5_ from CIL Isotopes (Massachusetts, USA). Palmitate-D_3_, linoleate-D_3_, stearate-D_3_, and palmitate-D_3_-^13^C_16_ were synthesized by Red Glead Discovery AB (Lund, Sweden). All standards were used without further purification (purity ≥95%).

Individual stock solutions of each analyte were prepared in acetone, in the case of ester forms, and in acetonitrile (ACN) for the rest of the compounds, in the range of 100–1000 mg/L and stored in the darkness at −18 °C. Working analyte mixtures were daily prepared by dilution with the appropriate volume of ACN.

All chemicals were of analytical reagent grade (unless otherwise indicated) and used as received. Heptane for chromatography and chloroform, ethyl acetate, isopropanol (IPA), acetone, and methanol (MeOH) of HPLC grade and ACN LC–MS grade were from VWR (Fontenay-sous-Boris, France). MeOH (LC–MS grade) was from J.T. Baker (Gliwice, Poland). Ammonium formate, an eluent additive for LC–MS (>99%), was from Sigma Aldrich Chemie and formic acid (LC–MS) grade from Fisher Chemical (Praha, Czech Republic). Ultrapure water (18.2 MΩ/cm) was generated with a 10A Millipore system (MA, USA).

### Apparatus and Software

The analysis of the target compounds was performed using an Agilent 1260 Infinity II SFC system coupled with a 6495 QqQ-mass spectrometer using both electrospray ionization (ESI) and atmospheric pressure chemical ionization (APCI) sources (Agilent Technologies, Waldbronn, Germany). The UHPSFC-MS system was controlled with the MassHunter Workstation Software v. 10.0 from Agilent Technologies. Data analysis was carried out using the same software. Separation of the vitamins was achieved on a Torus 1-aminoanthracene (1-AA) column (100 mm × 3.0 mm, 1.7 μm) with a Torus 1-AA pre-column (5 mm × 2.1 mm, 1.7 μm), both from Waters Chromatography. Experimental design was performed using the software Umetrics MODDE v. 12.1.0.3948 from Sartorius Stedim Biotech.

### Samples

In this study, human plasma samples were analyzed in order to demonstrate the applicability of the methodology for the determination of vitamin D and vitamin D metabolites in biological matrices. The collection of plasma samples was approved by the Regional Ethical Committees of Malmö/Lund. Samples were from fasted individuals and had been stored at −80 °C until analyses. The plasma samples were fasting samples from a study of obese individuals and included two females and three males, age 40–70 and BMI 32–36, (labeled H-1, H-2, H-3, H-4, and H-5). The concentration level of vitamin D in samples was calculated using a matrix-matched calibration curve. LOD and LOQ were determined via the weighted 1/*x*^2^ calibration curve as 3 and 10 times the standard deviation of the intercept for LOD and LOQ, respectively.

### Sample Extraction Procedure

The adjusted previously published method was used for extraction.^15,19^ Briefly, plasma samples (500 ± 0.1 μL) were placed in glass test tubes covered with aluminum foil to avoid analyte degradation.^1^ Then, 1250 μL of ACN was added to the sample and vortexed for 1 min to achieve protein precipitation. The mixture was centrifuged for 15 min at 2472*g* and 4 °C, after which the supernatant was collected. Then, the procedure was repeated again with 250 μL of ACN. Both supernatants were combined and evaporated to dryness under a nitrogen stream. After protein precipitation, 500 μL of ethyl acetate and 250 μL of ultrapure water were added to the dried sample, vortexed for 1 min, and centrifuged for 5 min at 3000 rpm and 4 °C. The organic layer was transferred into a new glass tube. The procedure was repeated for the remaining aqueous part by adding 500 μL of ethyl acetate. Finally, both organic layers were combined and evaporated under a nitrogen stream, and the residue was dissolved in 37.5 μL of ACN. The samples were stored in −80 °C until the analysis.

### UHPSFC-(QqQ)-MS/MS Determination

Optimal chromatographic conditions were obtained using a mobile phase consisting of CO_2_ (mobile phase A) and MeOH as a co-solvent (mobile phase B) and applying the following gradient: the initial composition of the mobile phase was 2% of B at a flow rate of 2.0 mL/min. It was maintained for 1.5 min. Then, B was changed to 5% in 0.25 min and maintained for 3.25 min. It was increased to 15% in 2 min and held for 1.50 min. Finally, the initial conditions were established in 1 min and maintained for 2 min. The total analysis time was 8.0 min. The column temperature was fixed at 50 °C and the backpressure at 190 bar while the injection volume was 6 μL at 5 °C using an overfeed volume of 4 μL and a feed speed of 400 μL/min.

Optimal ionization was achieved using APCI in positive mode. The capillary voltage was set at 3.75 kV, corona current at 5 μA, drying gas (N_2_) flow at 11 L/min, and its temperature at 175 °C. The vaporizer gas (N_2_) temperature was 362 °C, and the nebulizer gas pressure (N_2_) was 30 psi.

The MS system was operated in multiple-reaction monitoring (MRM) mode. MS/MS experiments were performed by fragmentation of the protonated molecule [M + H]^+^ that was selected as the precursor ion in most cases. Four identification points were used, i.e., one precursor, two product ions, and the retention time. A maximum tolerance of ±20% for the relative ion intensities of the product and precursor ions was tolerated.^20^

## Results and Discussion

### UHPSFC-(QqQ)-MS/MS

Initially, precursor ions and MS/MS fragmentation patterns of all target compounds were obtained under system default conditions using ESI and APCI sources (Table S1 in the Supporting Information). Here, analytes were directly infused in the system individually at a concentration of 2 mg/L. Scan spectra were acquired in both positive and negative modes using MeOH, 0.1% formic acid in MeOH, and 0.2% ammonium formate in MeOH as infusion solvents based on the conditions previously reported in the literature.^10,15^ Results indicated that positive mode provided better ionization for both ion sources since no signal was found for most of the analytes under negative mode (data not shown). Based on that, subsequent single ion monitoring (SIM), product ion (PI), and MRM studies were carried out with the aim to obtain MS/MS data of all target analytes in positive mode using both ESI and APCI. Additionally, collision energies in the range of 10–45 eV were also evaluated for each transition in order to achieve the highest sensitivity. The two most intense transitions for each compound were selected for further studies (see Table S2).

### Ionization Source and Make-Up Solvent Selection

Some authors have applied additional derivatization steps in order to enhance the detectability.^9,12,21^ However, the possibility of using a selective analyzer, such as the QqQ, in combination with an efficient ionization of the analytes, could reach high sensitivity, avoiding such additional steps. For this reason, once the transitions for each compound had been obtained, the limit of detections (LODs) achieved using both ion sources (APCI and ESI) were compared and the influence of different make-up solvents was thoroughly evaluated. The influence of make-up solvents for atmospheric pressure ionization sources has been reported to be of great importance.^22,23^ Based on the results obtained in previous screening experiments, a flow of 0.5 mL/min of 0.5% formic acid in MeOH, MeOH, and 0.2% ammonium formate in MeOH were evaluated, as well as the absence of a make-up solvent, under the UHPSFC conditions previously applied for a similar group of analytes with slight modifications.^15^Table 1 shows the LODs of some representative analytes, calculated as the concentration that provides a signal-to-noise ratio higher than 3 and obtained as a mean of three separate injections. On the one hand, for ESI, the highest detectability (lowest LOD) was obtained when no make-up solvent was used, except for ester forms. The esters elute at the beginning of the chromatographic separation when the mobile phase is 98% composed by CO_2_, which means that, after column outlet, the analytes have a relatively little co-solvent, enabling their transfer to the MS system. On the other hand, for APCI, the lowest LODs were reached when 0.5% of formic acid in MeOH was applied. This difference is related to the mass-flow dependence of APCI mode, in which the solvent plays a key role in the ionization process.^22,24^ Apart from that, the lowest LODs were in general achieved using the APCI, mainly attributed to the reduced background noise. Thus, APCI in positive mode using 0.5% of formic acid in MeOH was selected for analysis.

**Table 1:** Comparison of LODs for Representative Analytes Applying ESI and APCI Sources and Different Make-up Solvents^a^

	LOD ± SD (μg/L)					
make-up solvent	palmitate-D_3_	D_3_	25-OH-D_3_	1-OH-D_3_	24,25-(OH)_2_-D_3_	1,25-(OH)_2_-D_3_
ESI						
0.5% FA (v/v) in MeOH	9.63 ± 3.03	11.59 ± 5.45	104.9 ± 26.9	952.4 ± 95.6	161.7 ± 1.67	750.0 ± 233.8
MeOH	9.55 ± 0.80	9.06 ± 0.84	84.4 ± 25.4	1153 ± 200	422.5 ± 40.4	289.9 ± 77.6
0.2% AF (w/v) in MeOH	9.10 ± 4.43	9.30 ± 1.57	156.7 ± 42.1	1500 ± 259	177.9 ± 109.0	132.8 ± 3.9
without solvent	9.20 ± 2.32	1.30 ± 0.37	5.39 ± 0.24	20.9 ± 1.3	28.0 ± 10.4	42.4 ± 15.0
APCI						
0.5% FA (v/v) in MeOH	0.21 ± 0.10	0.25 ± 0.05	1.29 ± 0.34	1.34 ± 0.07	1.44 ± 0.72	2.33 ± 0.22
MeOH	0.33 ± 0.04	0.34 ± 0.05	3.00 ± 0.35	2.09 ± 0.40	1.59 ± 0.33	5.91 ± 1.32
0.2% AF (w/v) in MeOH	0.33 ± 0.10	0.82 ± 0.42	6.46 ± 0.52	1.23 ± 0.20	5.00 ± 0.16	7.19 ± 1.07
without solvent	1.46 ± 0.40	0.97 ± 0.14	6.45 ± 0.01	2.54 ± 0.50	10.8 ± 3.6	10.4 ± 7.3

a LOD: limit of detection, FA: formic acid, AF: ammonium formate. Ion source default conditions were applied (Table S1).

Additionally, keeping in mind the mentioned dependence of this ionization mode on the mass flow but also trying to decrease the consumption of organic solvents during the analysis procedure, a final study modifying the flow rate of the make-up solvent was also performed. Results, as shown in Figure S1, indicate that a make-up flow of 0.05 to 0.1 mL/min gives the highest detectability of all compounds, while a higher flow or no flow lowered the detectability. Hence, a flow of 0.1 mL/min was selected for further studies.

### Study of the Chromatographic Separation

**Figure 1:**
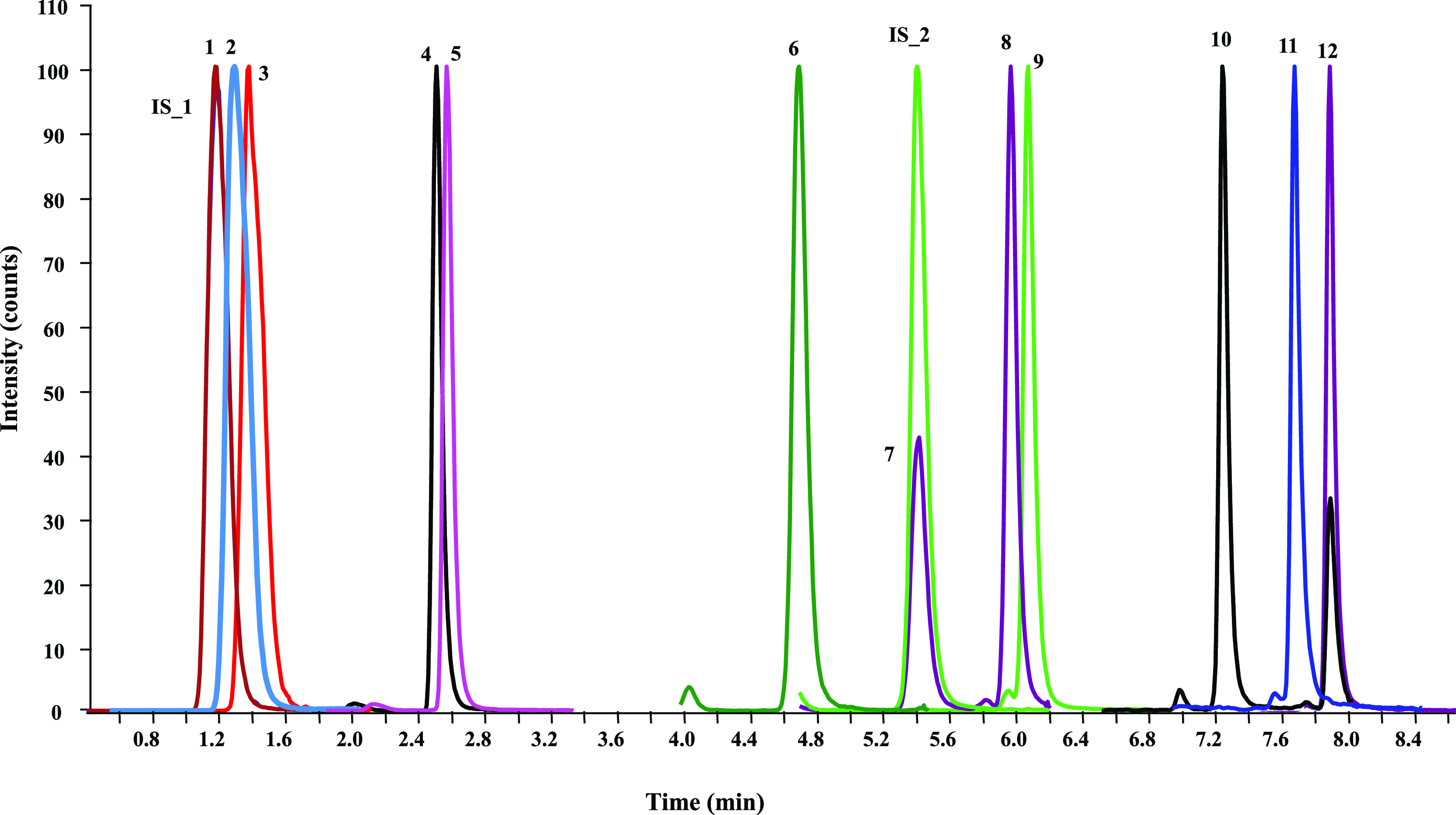
Normalized UHPSFC-(QqQ)-MS/MS chromatogram of the best separation achieved for all compounds under the chromatographic conditions described in the Experimental Section. Torus 1-aminoanthracene (1-AA) column at 50 °C using a mobile phase consisting of CO_2_ (mobile phase A) and MeOH as the co-solvent (mobile phase B). (1) Palmitate-D_3_; (IS_1) palmitate-D_3_-^13^C_16_; (2) stearate-D_3_; (3) linoleate-D_3_; (4) D_3_; (5) D_2_; (6) 25-OH-D_2_; (IS_2) 25-OH-D_3_-^13^C_5_; (7) 25-OH-D_3_; (8) 1-OH-D_3_; (9) 1-OH-D_2_; (10) 24,25-(OH)_2_-D_3_; (11) 1,25-(OH)_2_-D_2_; and (12) 1,25-(OH)_2_-D_3_.

After selecting the ion source and make-up solvent, the chromatographic separation was investigated with respect to resolution and peak capacity, considering all the selected vitamin D compounds, including several isomers. Based on a previous study in which vitamins D_2_ and D_3_ along with their hydroxylated forms were separated by UHPSFC, a column was selected (1-AA), as well as a preliminary gradient program.^15^ Initially, a variation in the percentage of the co-solvent between 3 and 15% in 7 min and a backpressure of 110 bar, flow rate of 2 mL/min, and temperature of 35 °C were applied. However, a complete separation of isomers 25-OH-D_3_ and 1-OH-D_3_ was not achieved under these conditions. Additionally, the introduction of ester forms in this work makes the separation more complex since the long hydrocarbon chains of these compounds provide them with very low polarity compared to the rest of the compounds (see Table S3). This fact brings about a challenge of both a wide range of polarities combined with the fact that some of the analytes are structural isomers. Hence, this requires a thorough evaluation of the chromatographic conditions that allows the comprehensive separation of all of them and with the shortest analysis time possible. In this respect, several modifications were carried out, not only on the mobile phase composition (the proportion of the co-solvent was varied between 2 and 15%) but also on the backpressure (varied between 110 and 220 bar) and temperature (35–55 °C), since these two other parameters also have an influence on the density of the mobile phase and, consequently, on the separation performance.^25^ The best separation in terms of resolution and peak capacity was obtained by applying the conditions described in the Experimental Section and in Figure 1, giving a short analysis time of 8.0 min.

### Optimization of MS/MS Conditions Using Design of Experiment (DoE)

**Figure 2:**
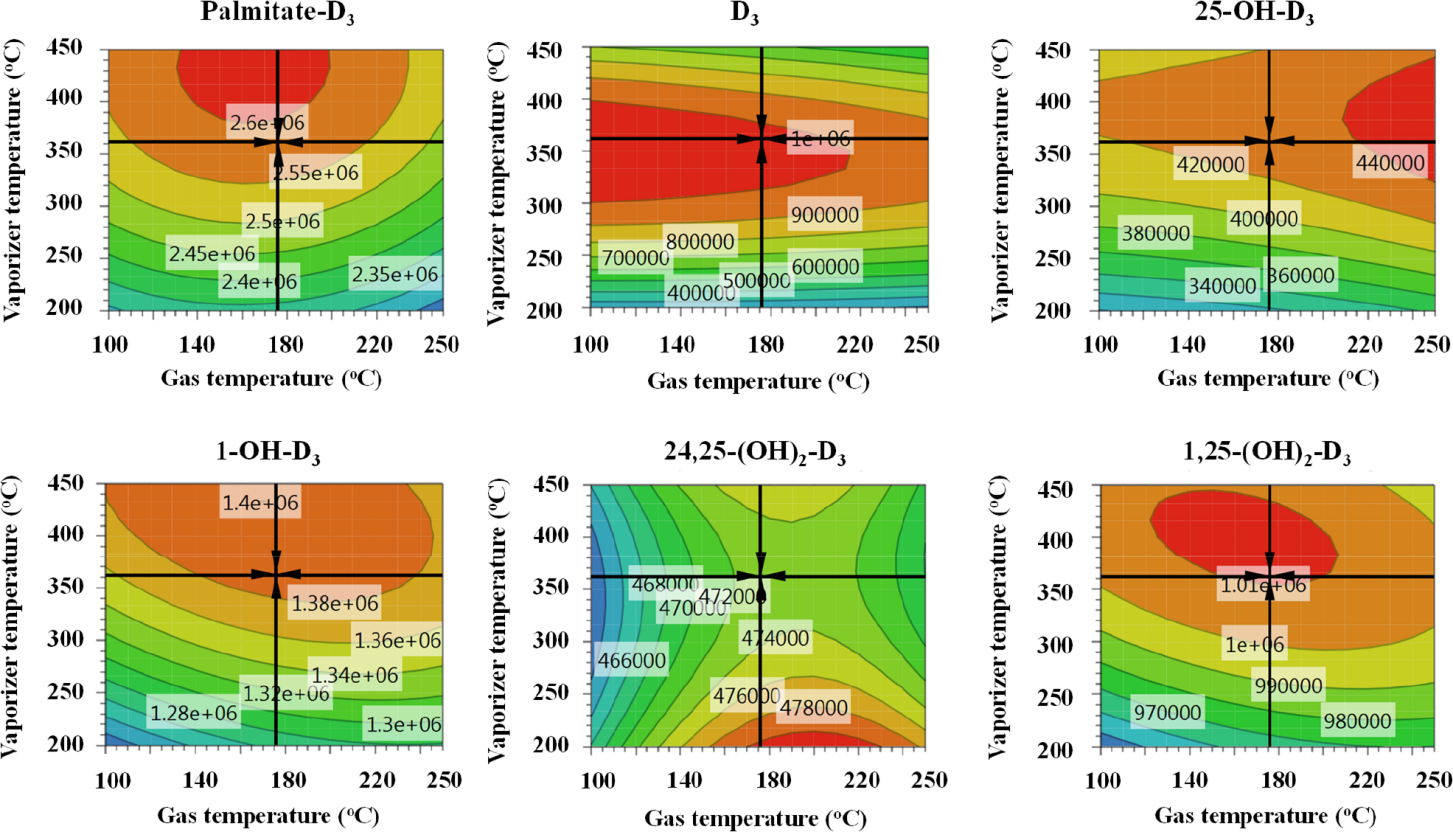
Contour plots of some representative compounds obtained from the RSM full factorial DoE for the optimization of the ionization source parameters for APCI. The variation of the areas, taking into account the modification of the vaporizer temperature and source gas temperature, around the optimal point (center of the black cross) is represented for each analyte. Conditions are described in the Experimental Section. Fixed conditions: capillary voltage: 3.75 kV; corona current: 5 μA; nebulizer gas pressure (N_2_): 30 psi.

Once the most adequate ion source and separation conditions of the selected group of analytes had been selected, a thorough evaluation of MS conditions was carried out. In this respect, those parameters that could affect their determination in terms of selectivity and sensitivity were optimized applying a DoE. Initially, a screening study was done with the aim to select the most influential parameters and reducing the complexity of the study. The capillary voltage (2000–5500 V), gas source temperature (100–250 °C), drying gas (N_2_) flow (11–20 L/min), nebulizer pressure (10–50 psi), vaporizer temperature (200–450 °C), and corona current (2–8 μA) were evaluated in the ranges indicated using a fractional factorial DoE with 19 experiments and 3 central points. Results indicated that the gas source temperature, drying gas flow, and vaporizer temperature had the highest influence on the majority of selected analytes (data not shown). For this reason, these three parameters were selected to carry out response surface modeling using a full factorial design with three levels, 32 experiments, and 5 central points, fixing the rest of the parameters (capillary voltage: 3.75 kV; corona current: 5 μA; nebulizer gas pressure (N_2_): 30 psi). The fitting of the model was good for all analytes with an adequate significance (*R*^2^ > 50%), good prediction precision (*Q* > 40%), good model validity (>30%), and adequate reproducibility (>50%) for all compounds. The optimal point was found when the vaporizer gas temperature was set at 362 °C, the drying gas flow at 11 L/min, and the gas source temperature at 175 °C. As can be seen in the contour plots in Figure 2, the variation of the area obtained for targeted compounds is presented based on the modification of two of the most influential factors, i.e., the vaporizer gas temperature and gas source temperature; the optimal point provided the largest peak area for most of them (red and orange zones), except for 24,25-(OH)_2_-D_3_. In order to select a situation of compromise that provided the highest detectability for most analytes, these conditions were selected for the analysis of these compounds.

### UHPSFC-(QqQ)-MS/MS Validation

As this constitutes the first work in which an analytical methodology has been developed for the simultaneous separation and determination of vitamin D and its D hydroxylated and ester metabolites, a careful validation was carried out in order to test the suitability of the analytical approach and guarantee the reliability of the results obtained from its application.

Detectability of the new method was evaluated by obtaining the instrumental LODs and LOQs as those concentrations that provided a signal-to-noise ratio higher than 3 and 10, respectively, from three consecutive injections. LODs, shown in Table 2, were lower than 0.91 μg/L and LOQs below 3.03 μg/L, which were six times lower than the ones obtained previously for similar groups of compounds using SFC in combination with MS and quadrupole-time-of-flight (QToF) spectrometry^15^ and similar or slightly higher than other studies using the same analyzer (QqQ).^16^ However, in both these cases, a smaller group of compounds was analyzed and a longer analysis time was necessary to accomplish the separation. A comparison regarding ester forms is not possible because there are no published results for such compounds up to date.

**Table 2:** Instrumental and Matrix-Matched Calibration Data of the Selected Compounds^e^

		instrumental calibration			matrix-matched calibration		
analyte	retention time (min)	LOD^a^ (μg/L)	LOQ^a^ (μg/L)	LOQ^c^ literature (μg/L)	LOD_method_^b^ (μg/L)	LOQ_method_^b^ (μg/L)	LOQ_method_ literature^d^ (μg/L)
palmitate-D_3_	1.20	0.19 ± 0.05	0.64 ± 0.16		2.68	8.13	
stearate-D_3_	1.30	0.07 ± 0.01	0.23 ± 0.07		2.94	8.92	
linoleate-D_2_	1.40	0.16 ± 0.07	0.52 ± 0.24		1.01	3.05	
D_3_	2.53	0.16 ± 0.01	0.53 ± 0.03	0.087–5.43	0.21	0.65	1.00–2.00
D_2_	2.60	0.22 ± 0.06	0.75 ± 0.21	0.092–7.25	0.20	0.60	1.00–2.00
25-OH-D_2_	4.70	0.34 ± 0.02	1.12 ± 0.06	0.095–17.22	0.19	0.57	1.00–4.00
25-OH-D_3_	5.40	0.88 ± 0.14	2.93 ± 0.45	0.077–6.56	2.33	7.06	1.00–4.00
1-OH-D_3_	5.98	0.40 ± 0.06	1.34 ± 0.19	6.56	0.14	0.41	
1-OH-D_2_	6.07	0.17 ± 0.01	0.55 ± 0.01	18.11	0.10	0.29	
24,25-(OH)_2_-D_3_	7.26	0.25 ± 0.02	0.84 ± 0.08	0.272–1.19	0.16	0.48	1.00–1.30
1,25-(OH)_2_-D_2_	7.69	0.60 ± 0.04	2.01 ± 0.13	0.704–6.18	0.13	0.40	
1,25-(OH)_2_-D_3_	7.90	0.29 ± 0.04	0.98 ± 0.14	0.635–7.57	0.21	0.63	

a The concentration that provides a signal-to-noise ratio higher than 3 and 10 for LOD and LOQ, respectively.

b Determined via the calibration curve as 3 and 10 times the standard deviation of the intercept for LOD_method_ and LOQ_method_, respectively. Palmitate-D_3_-^13^C_16_ was used as surrogate for ester metabolites and 25-OH-D_3_-^13^C_5_ for the rest of the compounds.

c Data obtained from Jumaah et al. and Liu et al.^15,16^

d Data obtained from Zhang et al., Gervasoni et al., Adamec et al., Zelzer et al., Mochizuki et al., and Abouzid et al.^11−13,21,26,27^

e Palmitate-D_3_-^13^C_16_ was used as the IS for ester metabolites and 25-OH-D_3_-^13^C_5_ for the rest of the compounds.

In the same way, calibration curves for all analytes were prepared by injecting seven different concentrations in the range of 1.0–500 μg/L (except for 25-OH-D_3_, 25-OH-D_2_, 1-OH-D_3_, and 1,25-(OH)_2_-D_2_, for which started at 2.5 or 5 μg/L) using 25-OH-D_3_-^13^C_5_ as the internal standard (IS) for vitamin and hydroxylated analogues and palmitate-D_3_-^13^C_16_ for ester metabolites. Determination coefficients (*R*^2^) higher than 0.9992 were obtained in all cases, indicating the linearity of the method in the range of concentrations studied (see Table S4). Additionally, with the aim of testing the repeatability of the methodology in terms of retention times (*t*_R_) and peak areas, precision was evaluated intraday, by injecting 6 times, three different levels at the low, medium, and high concentrations (5, 250, and 500 μg/L), and interday, repeating the study in three different days. Relative standard deviations (RSD, %) obtained for *t*_R_, as shown in Table S5, were lower than 2.0% for both intra- and interday precision, as well as below 10% for peak areas, without IS correction. These results demonstrated a good repeatability of the developed instrumental method.

### Application of the Methodology to the Analysis of Plasma Samples

Vitamin D metabolites were extracted from human plasma according to the previously published method.^15,19^ Protein precipitation was carried out as the first step to release vitamin D metabolites from the proteins. Indeed, vitamin D metabolites are strongly bound to transport proteins, including vitamin D binding protein, which transports 95–99% of all the vitamin D metabolites in plasma, and albumin and lipoproteins, transporting 1–5% of the vitamin D metabolites. Only negligible amounts occur in the free form.^28^ After protein precipitation, a liquid–liquid extraction method originally developed for extraction of mono- and dehydroxylated vitamin D forms was applied.^19^

### Partial Validation: Linearity, Matrix Effect, and Recovery Evaluation

First, linearity, LOD, and LOQ were evaluated for all compounds. The matrix-matched calibration curve from human plasma spiked by analytes was prepared and extracted. The applied method showed good linearity with *R*^2^ higher than 0.9606 for all compounds (Table S6), as well as acceptable detectability with LOQs in the range of 0.29–8.92 μg/L (see Table 2). No carry over was observed in blank samples after the injection of the highest concentration level 150 μg/L (data not shown). LOD and LOQ values are in the same order of magnitude as those obtained for similar matrices in previous published reports for the analysis of D_3_, D_2_, and hydroxylated metabolites, including blood and serum samples and using chromatographic techniques in combination with MS and QqQ as the analyzer.^11−13,21,26,27^ However, in all these published studies, a fewer number of compounds (maximum: four) were evaluated simultaneously and derivatization steps were used, thereby increasing the complexity of the procedure.^12,21^

Matrix effects were studied following the Matuszewski method^29^ by comparing the peak areas obtained for spiked samples at the end of the extraction procedure and for standards at the same concentration. In order to increase the selectivity and correlate the results, two stable isotopically labeled ISs were added to all the samples of the matrix-matched calibration (MMC): palmitate-D_3_-^13^C_16_ for ester forms and 25-OH-D_3_-^13^C_5_ for the other compounds. As can be observed in Table S6, no significant matrix effects were found in most cases with values in the range of 83–112% with RSD < 7%. Only for 1,25-(OH)_2_-D_3_ at 15 μg/L and for D_2_ and D_3_ at 150 μg/L concentration levels, a slight signal suppression was found (76–79%). A slight enhancement for 25-OH-D_3_ (>120%) was observed for both tested concentration levels, which can be caused by the natural presence of the compound in human plasma. It is not an ideal situation with only one isotopically IS for all hydroxylated compounds, as well as vitamin D molecules, but due to the high price and unavailability in the market, it was not possible to obtain the IS for each analyzed compound.

A recovery study was carried out by five replicates at two different levels of concentration (15 and 150 μg/L). Relative recovery values correlated to ISs differed for the different vitamin D metabolites. As mentioned before, the method used for the plasma samples was originally optimized for only five hydroxylated compounds, 25-OH-D_2_, 25-OH-D_3_, 24,25-(OH)_2_-D_3_, 1,25-(OH)_2_-D_2_, and 1,25-(OH)_2_-D_3_, where the recovery was in the range of 97–111% with RSD < 13%. Only for 25-OH-D_3_, the recovery was higher at the lower concentration level (157%), as well as at the higher concentration level (125%), which could be due to its high abundance in human plasma. For 1-OH-D_3_ and 1-OH-D_2_, the recovery was slightly lower, in the range of 61–74% with RSD < 12% for both concentration levels. Vitamins D_2_ and D_3_ provided recovery values in the range of 35–51% with RSD < 17%. The lower relative recovery could be caused by different absolute recoveries of metabolites and ISs. A different situation was observed for the esters. The recovery range was wider, 56–215% with RSD < 13%, and a large difference for the different concentration levels was observed. These results can be explained by the very poor extraction recovery without IS compensation, which was <0.7% for all esters, as well as by the different physicochemical properties of esters compared to hydroxylated vitamin D forms. All results are summarized in Table S6.

### Analysis of Human Plasma Samples

**Figure 3:**
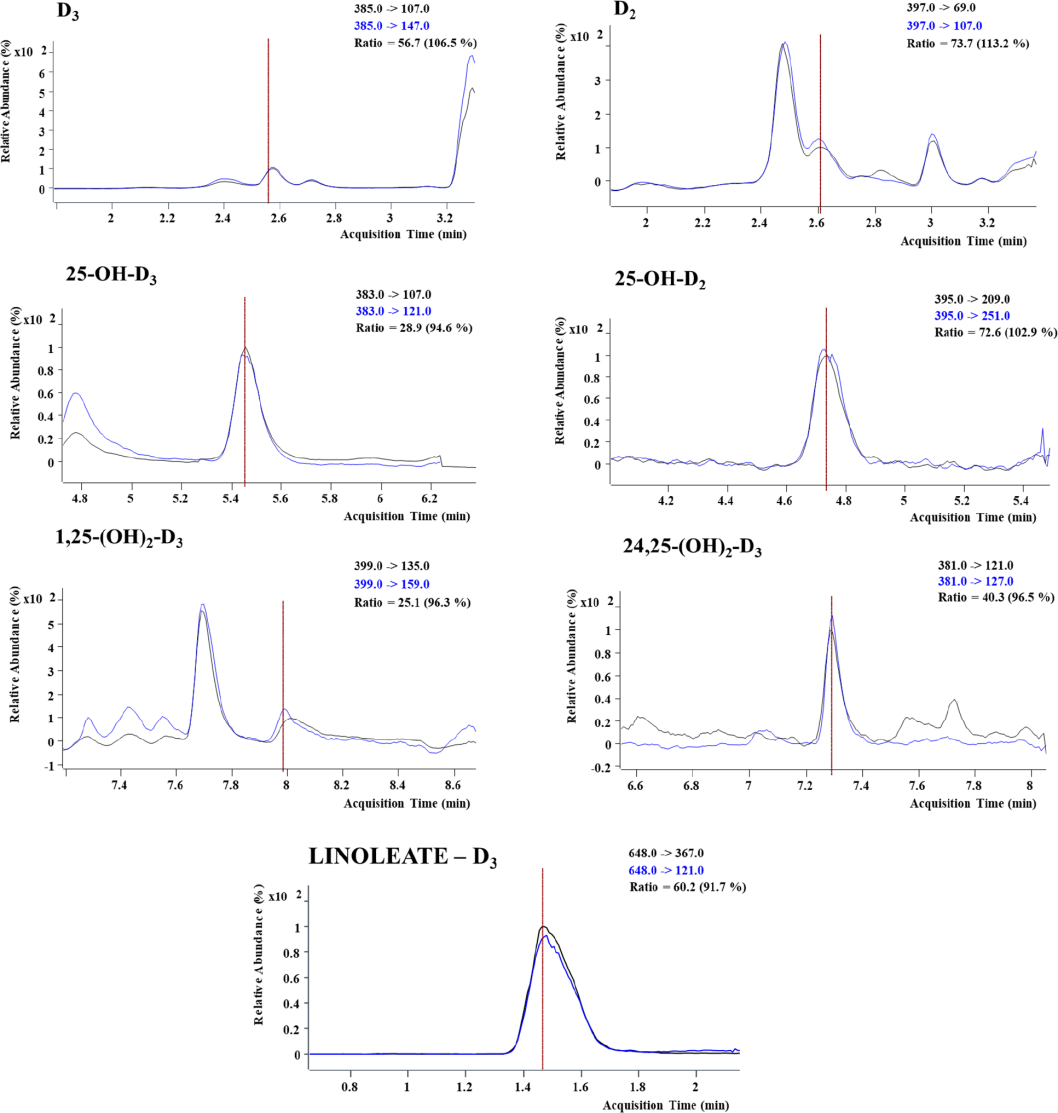
Examples of UHPSFC-(QqQ)-MS/MS extracted ion chromatograms of vitamin D_3_, D_2_, and various metabolites from a blood plasma sample under the chromatographic conditions described in the Experimental Section. Torus 1-aminoanthracene (1-AA) column at 50 °C using a mobile phase consisting of CO_2_ (mobile phase A) and MeOH as the co-solvent (mobile phase B).

Based on the promising results achieved from the validation study, the methodology was applied to the analysis of human plasma samples from five obese, but otherwise essentially healthy, individuals (Table 3). Preliminary results indicated the presence of D_3_ and D_2_ in all analyzed samples, as well as the metabolite 25-OH-D_2_, which constitutes the most stable form of this group of compounds in the blood^1^ (Figure 3), and dihydroxylated forms, 24,25-(OH)_2_-D_3_ and 1,25-(OH)_2_-D_3_, as well as linoleate-D_3_.

**Table 3:** Results of the Analysis of Human Plasma Samples Applying the Developed Method^a^

human sample concentration (μg/L)							
sample	linoleate- D_3_	D_3_	D_2_	25-OH-D_2_	25-OH-D_3_	24,25-(OH)_2_-D_3_	1,25-(OH)_2_-D_3_
H-1	detected	1.97 ± 0.29	<LOD	0.63 ± 0.63	<LOD	1.62 ± 1.00	1.51 ± 0.63
H-2	detected	8.56 ± 0.35	<LOD	detected	<LOD	1.20 ± 1.00	1.26 ± 0.63
H-3	detected	2.52 ± 0.29	<LOD	detected	<LOD	0.74 ± 0.99	1.09 ± 0.63
H-4	detected	0.74 ± 0.28	detected	detected	<LOD	0.79 ± 1.00	1.21 ± 0.63
H-5	detected	3.65 ± 0.30	detected	detected	<LOD	1.10 ± 1.00	0.88 ± 0.62

a Detected = compound is detected (>LOD, <LOQ). <LOD = not detected. *n* = 3.

However, the concentrations of 25-OH-D_3_ were found under the LOD (2.33 μg/L), so the presence of the compound could not be confirmed in the sample. 25-OH-D_2_ concentrations were lower than the LOQ (0.57 μg/L), except in sample H-1. The concentration observed under the LOQ could be related to the fact that the evaluation of 25-OH-D_2_ is usually carried out in serum,^1,30^ probably because the plasma samples contain anticoagulant factors that make this matrix more complex.^31^ The concentration calculated for linoleate-D_3_ was very close to the LOQ (3.05 μg/L) for all samples, which was also the case for D_2_ in two samples. For vitamin D_3_, 24,25-(OH)_2_-D_3_, and 1,25-(OH)_2_-D_3_, however, the concentration was higher than LOQ in all samples.

The absolute values for D_3_, D_2_, 25-(OH)-D_2_, and 24,25-(OH)_2_-D_3_ are reasonable, although they are on the low side of what has been reported for these metabolites. This could be due to the fact that the samples were from obese individuals since obesity is associated with low vitamin D levels (5), presumably due to trapping of vitamin D in adipose tissue. 1,25-(OH)_2_-D_3_, on the other hand, was higher than expected. Linoleate-D_3_ has, to our knowledge, never been reported for human plasma samples. Linoleate-D_3_ and other vitamin D esters presumably occur in the bloodstream as constituents of lipoprotein particles, although the major part of vitamin D esters in the body is expected to be found in the adipose tissue. 25-(OH)-D_3_, which is normally the major form of vitamin D in the circulation and the metabolite analyzed in the clinic to assess vitamin D status, was found in concentrations under the LOD (2.33 μg/L), so the detection of this metabolite could not be accomplished. The reason for the low levels of 25-(OH)-D_3_ and the high levels of 1,25-(OH)_2_-D_3_ is unknown and deserves further investigations, including studies comparing plasma and serum samples and studies of the optimal storage conditions for samples to be profiled for vitamin D metabolites.

These results showed the suitability of the developed methodology for the analysis of these type of matrices. However, further effort should be done in future work to improve the sensitivity of the methodology, especially for the determination of the ester forms, for which no data have been reported before in the literature. Consequently, there are no references for the levels of these compounds in the biological samples.

## Conclusions

In this work, a novel and comprehensive analytical methodology has been developed that for the first time allows the simultaneous separation and determination of vitamins D_3_ and D_2_ as well as their hydroxylated and ester analogues using UHPSFC-(QqQ)-MS/MS with a short analysis time of 8.0 min. The comparison between ESI and APCI showed a higher sensitivity for the whole group of analytes studied when APCI in positive mode was used. The method was successfully validated, obtaining good sensitivity, as well as excellent linearity and intra- and interday precision_._

Based on the promising results obtained, the methodology was applied to the analysis of plasma samples. The whole procedure was validated, obtaining good extraction efficiency, reproducibility, and adequate sensitivity. The analysis of human samples from different individuals was also carried out. Results showed the potential of the developed methodology for its application in biological samples, which constitutes the first step to acquiring knowledge about the role that minoritary vitamin D metabolites play in physiological processes. In this respect, the present work represents the first analytical methodology for the analysis of ester forms as well as their simultaneous evaluation together with other vitamin D analogues with varied polarities developed up to date.

## Supporting Information Available

(Table S1) Default ion source conditions applied during optimization; (Table S2) MS/MS transitions and collision energies selected for the target compounds and internal standard determination; (Figure S1) comparison of obtained peak areas at different make-up solvent flow rates; (Table S3) structures and properties of vitamin D analogues; (Table S4) instrumental calibration data of the selected compounds; (Table S5) intra- and interday precision of retention times (*t*_R_) and peak areas at different concentrations (levels I–III) for the developed UHPSFC-(QqQ)-MS/MS method; and (Table S6) matrix-matched calibration data of the selected compounds in spiked plasma samples at two concentration levels (PDF)
