# Supplementary material for: Simultaneous Determination of Vitamin D and Its Hydroxylated and Esterified Metabolites by Ultrahigh-Performance Supercritical Fluid Chromatography–Tandem Mass Spectrometry
Source: Anal Chem. 2022 Feb 9;94(7):3065–73. doi: 10.1021/acs.analchem.1c04016 (PMC8867463; doi:10.1021/acs.analchem.1c04016)
Supplement: Supplementary file 1 — ac1c04016_si_001.pdf [file ac1c04016_si_001.pdf]

Supporting Information:

Simultaneous determination of vitamin D and its hydroxylated and esterified metabolites by ultrahigh-performance supercritical fluid chromatography - tandem mass spectrometry

Bárbara Socas-Rodríguez[1], Veronika Pilarová[1,2], Margareta Sandahl[1], Cecilia Holm[3], Charlotta Turner[1,*]

[1]Department of Chemistry, Centre for Analysis and Synthesis, Lund University, P.O. Box 124, 22100 Lund, Sweden.

[2]Department of Analytical Chemistry, Faculty of Pharmacy in Hradec Králové, Charles University, Akademika Heyrovského 1203, 500 05 Hradec Králové, Czech Republic

[3]Department of Experimental Medical Science, Faculty of Medicine, Lund University, P.O. Box 124, 22100 Lund, Sweden.

*Corresponding author: +46 46 222 8125, Charlotta.Turner@chem.lu.se, https://orcid.org/0000-0001-9466-1149

Table of Content

Table S1 – Default ion source conditions applied during the optimization 2

Table S2 – MS/MS transitions and collision energies selected for the target compounds and internal standards determination 3

Figure S1 – Comparison of obtained peak areas at different make-up solvent flow rates......... 4

Table S3 – Structures and properties of vitamin D analogues 5

Table S4 – Instrumental calibration data of the selected compounds 8

Table S5 – Intra-day and inter-day precision of retention times (t_r) and peak areas at different concentrations (levels I-III) for the developed UHPSFC-(QqQ)-MS/MS method 9

Table S6 – Matrix matched calibration data of the selected compounds in spiked plasma samples at two concentration levels 10

Table S1 – Default ion source conditions applied during the optimization

Parameter	ESI (+/-)	APCI (+/-)
**Gas temp. (°C)**	200	120
**Vaporiser (°C)**	-	350
**Gas flow (L/min)**	14	14
**Nebuliser (psi)**	20	20
**Capillary voltage (V)**	3500 (+), 3000 (-)	4500 (+), 4500 (-)
**Corona (µA)**	-	4 (+), 10 (-)

Table S2 – MS/MS transitions and collision energies selected for the target compounds and internal standards determination

Analyte	MW (g/mol)	Precursor ion (m/z)	Product ion (m/z)	Collision energy (eV)	Analyte	MW (g/mol)	Precursor ion (m/z)	Product ion (m/z)	Collision energy (eV)
Palmitate-D_3	623.6	624	367	10	25-OH-D_3	400.6	383	107	25
			121	10				121	25
Palmitate-D_3-$^{13}C_{16}$ (*)	639.1	639	367	10	25-(OH)-D_3-$^{13}C_5$ (*)	405.6	388	159	25
			259	15				211	25
Stearate-D_3	651.1	651	367	10	1-OH-D_3	400.7	383	95	20
			259	15				107	20
Linoleate-D_3	647.1	648	367	20	1OH-D_2	412.7	395	69	20
			121	20				159	20
D_3	384.7	385	107	25	24,25-$(OH)_2$-D_3	416.7	381	121	20
			147	25				127	20
D_2	396.7	397	107	25	1,25-$(OH)_2$-D_2	428.7	411	83	20
			69	25				159	20
25-OH-D_2	412.7	395	209	25	1,25-$(OH)_2$-D_3	416.7	399	135	20
			251	25				159	20

(*) Internal standards

Figure S1 – Comparison of obtained peak areas at different make-up solvent flow rates

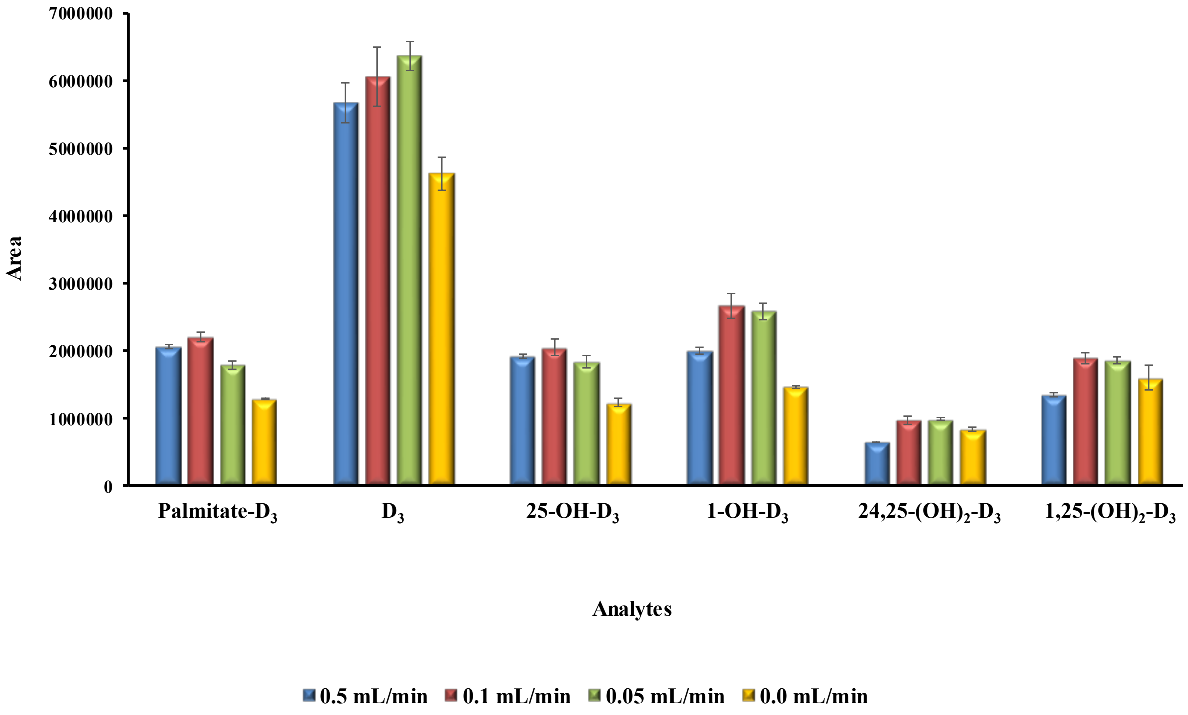

Figure S1.- Comparison of obtained peak areas using 0.5% of FA in methanol as make-up solvent at flow rates of 0.5, 0.1, 0.05 and 0 mL/min respectively, and using APCI at default ion source conditions (see Table S1).

Table S3 – Structures and properties of vitamin D analogues

Name	Structure	Molecular formula	Molecular weight (g/mol)	LogP	pKa	Melting point (°C)
Palmitate-D_3	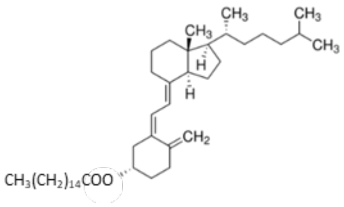	$C_{43}H_{54}O_2$	623.046	-	-	-
Stearate-D_3	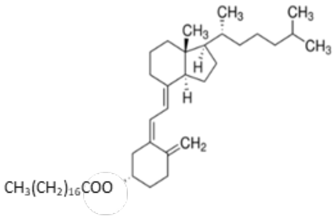	$C_{45}H_{78}O_2$	651.12	-	-	-
Linoleate-D_3	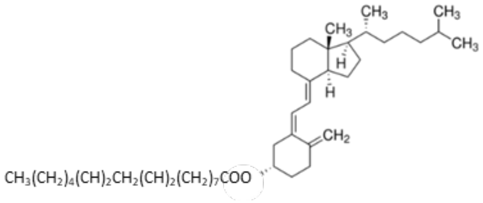	$C_{45}H_{54}O_2$	647.07	-	-	-
D_3	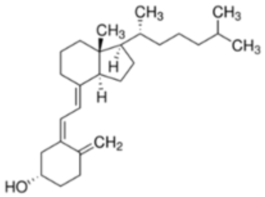	$C_{27}H_{44}O$	384.65	7.13 (a)	18.38;-1.3	84.5 (b)

D_2	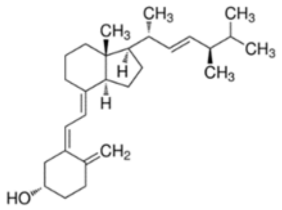	$C_{28}H_{44}O$	396.66	8.89(b)	6.35(b)	115-117 (b)
25-OH-D_2	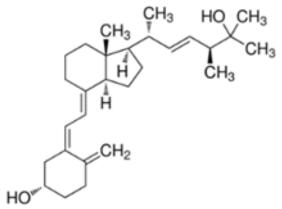	$C_{28}H_{44}O_2$	412.65	6(c)	-	-
25-OH-D_3	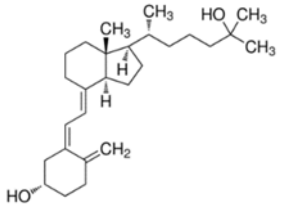	$C_{27}H_{44}O_2$	400.64	5.65(a)	18.38;-0.98(a)	--
1-OH-D_3	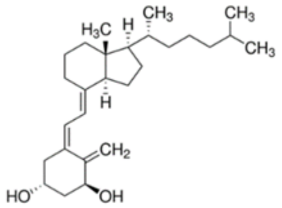	$C_{27}H_{44}O_2$	400.64	5.82	14.39;-2.8 (a)	136(b)
1-OH-D_2	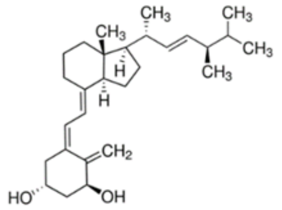	$C_{28}H_{44}O_2$	412.65	5.75 (a)	14.39, -2.8 (a)	-

24,25-$(OH)_2$-D_3	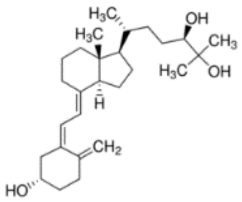	$C_{27}H_{44}O_3$	416.64	5(c)	-	-
1,25-$(OH)_2$-D_2	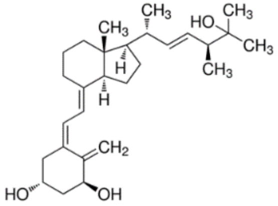	$C_{28}H_{44}O_3$	428.70	4.35(a)	14.39; -1(a)	-
1,25-$(OH)_2$-D_3	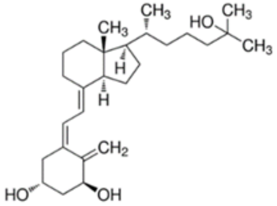	$C_{27}H_{44}O_3$	416.64	5(b)	14.39;-1.3	113-114(b)

a) Predicted data ChemAxon (https://docs.chemaxon.com). Taken from Pubchem (https://pubchem.ncbi.nlm.nih.gov); b) Experimental data. Taken from (https://www.drugbank.ca); c) Predicted data taken from PubChem (https://pubchem.ncbi.nlm.nih.gov).

Table S4 – Instrumental calibration data of the selected compounds

Analyte	Retention time	Concentration range (µg/L)	Calibration data (n = 7)		
			Slope ± S_a	Intercept ± S_b	R^2
Palmitate-D_3	1.20	1.0-500	$2.51 \cdot 10^{-4} \pm 2.44 \cdot 10^{-6}$	$-1.65 \cdot 10^{-4} \pm 6.41 \cdot 10^{-4}$	0.9999
Stearate-D_3	1.30	1.0-500	$2.60 \cdot 10^{-3} \pm 3.79 \cdot 10^{-5}$	$-7.70 \cdot 10^{-3} \pm 9.52 \cdot 10^{-3}$	0.9998
Linoleate-D_3	1.40	1.0-500	$1.60 \cdot 10^{-4} \pm 4.38 \cdot 10^{-6}$	$-5.04 \cdot 10^{-4} \pm 1.10 \cdot 10^{-3}$	0.9994
D_3	2.53	1.0-500	$1.38 \cdot 10^{-2} \pm 3.81 \cdot 10^{-4}$	$-6.23 \cdot 10^{-2} \pm 9.58 \cdot 10^{-2}$	0.9994
D_2	2.60	1.0-500	$1.19 \cdot 10^{-2} \pm 3.76 \cdot 10^{-4}$	$-6.01 \cdot 10^{-2} \pm 9.45 \cdot 10^{-2}$	0.9992
25-OH-D_2	4.70	2.5-500	$2.36 \cdot 10^{-3} \pm 6.58 \cdot 10^{-5}$	$-6.93 \cdot 10^{-3} \pm 1.45 \cdot 10^{-2}$	0.9994
25-OH-D_3	5.40	5.0-500	$4.25 \cdot 10^{-3} \pm 1.40 \cdot 10^{-4}$	$-2.21 \cdot 10^{-2} \pm 3.60 \cdot 10^{-2}$	0.9992
1-OH-D_3	5.98	2.5-500	$6.04 \cdot 10^{-3} \pm 1.07 \cdot 10^{-4}$	$-2.49 \cdot 10^{-2} \pm 2.70 \cdot 10^{-2}$	0.9998
1-OH-D_2	6.07	2.5-500	$9.97 \cdot 10^{-3} \pm 2.50 \cdot 10^{-4}$	$-4.15 \cdot 10^{-2} \pm 6.27 \cdot 10^{-2}$	0.9995
24,25-$(OH)_2$-D_3	7.26	1.0-500	$2.05 \cdot 10^{-3} \pm 6.07 \cdot 10^{-5}$	$-6.59 \cdot 10^{-3} \pm 1.53 \cdot 10^{-2}$	0.9993
1,25-$(OH)_2$-D_2	7.69	2.5-500	$1.51 \cdot 10^{-3} \pm 3.96 \cdot 10^{-5}$	$-4.89 \cdot 10^{-3} \pm 1.02 \cdot 10^{-2}$	0.9995
1,25-$(OH)_2$-D_3	7.90	1.0-500	$4.28 \cdot 10^{-3} \pm 1.09 \cdot 10^{-4}$	$-1.88 \cdot 10^{-2} \pm 2.73 \cdot 10^{-2}$	0.9995

R^2: Determination coefficient. S_a and S_b: Standard deviations of slope and intercept, respectively. Palmitate-D_3-$^{13}C_{16}$ was used as IS for ester metabolites and 25-OHD_3-$^{13}C_5$ for the rest of compounds.

Table S5 – Intra-day and inter-day precision of retention times (t_r) and peak areas at different concentrations (levels I-III) for the developed UHPSFC-(QqQ)-MS/MS method

Analyte	Level I		Level II		Level II	
	**Intra-day precision (RSD, %) (n = 6)**					
	t_r	Peak area	t_r	Peak area	t_r	Peak area
Palmitate-D_3	1.66	10.00	0.63	2.80	0.48	1.82
Stearate-D_3	1.64	3.78	0.55	1.99	0.53	1.06
Linoleate-D_3	2.04	9.77	0.73	4.85	0.82	2.81
D_3	0.09	5.82	0.11	2.48	0.09	1.97
D_2	0.14	7.73	0.00	2.15	0.08	1.48
25-OH-D_2	0.12	5.57	0.00	2.68	0.10	1.37
25-OH-D_3	0.19	5.91	0.08	3.33	0.10	1.34
1-OH-D_3	0.06	9.42	0.00	3.60	0.00	3.39
1-OH-D_2	0.08	8.42	0.00	3.88	0.00	1.40
24,25-$(OH)_2$-D_3	0.03	2.59	0.03	2.75	0.03	3.27
1,25-$(OH)_2$-D_2	0.03	7.72	0.00	2.77	0.00	3.02
1,25-$(OH)_2$-D_3	0.03	6.72	0.03	3.24	0.03	2.87

Analyte	Level I		Level II		Level III	
	**Inter-day precision (RSD, %) (n = 18)**					
	t_r	Peak area	t_r	Peak area	t_r	Peal area
Palmitate-D_3	1.88	9.52	0.78	4.76	0.82	3.18
Stearate-D_3	2.07	9.07	1.05	7.15	0.66	7.11
Linoleate-D_3	2.04	10.00	0.82	6.49	0.73	3.59
D_3	0.41	4.99	0.28	2.05	0.24	2.00
D_2	0.43	7.14	0.28	1.97	0.23	1.85
25-OH-D_2	0.56	5.92	0.41	2.91	0.38	1.99
25-OH-D_3	0.60	5.00	0.43	2.97	0.42	2.28
1-OH-D_3	0.37	6.34	0.28	3.10	0.25	3.54
1-OH-D_2	0.33	6.00	0.26	4.32	0.24	2.42
24,25-$(OH)_2$-D_3	0.18	4.90	0.12	2.37	0.11	2.69
1,25-$(OH)_2$-D_2	0.14	5.92	0.09	3.03	0.09	2.27
1,25-$(OH)_2$-D_3	0.11	4.29	0.09	3.67	0.09	2.65

Level I: 5 µg/L; Level II: 250 µg/L; Level III: 500 µg/L.

Table S6 – Matrix matched calibration data of the selected compounds in spiked plasma samples at two concentration levels

Analyte	Matrix effects (n = 5)[a)]		Recovery study (n = 5)		Matrix matched calibration data (n=7)			
	Level 1[a)] Matrix effects, % (RSD, %)	Level 2[a)] Matrix effects, % (RSD, %)	Level 1[b)] Recovery, % (RSD, %)	Level 2[c)] Recovery, % (RSD, %)	Concentration range (µg/L)	Slope ± S_a	Intercept ± S_b	R^2
Palmitate-D_3	101 (2)	101 (3)	144 (7)	83 (9)	10.0-150	$7.79\cdot10^{-3} \pm 6.33\cdot10^{-4}$	$-4.55\cdot10^{-4} \pm 6.33\cdot10^{-3}$	0.9606
Stearate-D_3	95 (3)	94 (2)	94 (6)	56 (13)	2.5-150	$7.70\cdot10^{-3} \pm 6.30\cdot10^{-4}$	$-8.48\cdot10^{-4} \pm 6.31\cdot10^{-3}$	0.9630
Linoleate-D_3	108 (5)	99 (5)	205 (7)	148 (12)	10.0-150	$3.68\cdot10^{-2} \pm 1.02\cdot10^{-2}$	$3.68\cdot10^{-2} \pm 1.02\cdot10^{-2}$	0.9783
D_3	90 (6)	78 (18)	45 (15)	47 (17)	1.0-150	$2.35\cdot10^{-2} \pm 1.40\cdot10^{-3}$	$5.71\cdot10^{-4} \pm 1.54\cdot10^{-3}$	0.9947
D_2	98 (5)	76 (13)	35 (9)	51 (13)	1.0-150	$2.61\cdot10^{-2} \pm 1.43\cdot10^{-3}$	$-1.38\cdot10^{-3} \pm 1.57\cdot10^{-3}$	0.9959
25-OH-D_2	113 (7)	111 (2)	98 (8)	103 (3)	1.0-150	$1.04\cdot10^{-2} \pm 5.40\cdot10^{-4}$	$-1.87\cdot10^{-4} \pm 5.92\cdot10^{-4}$	0.9947
25-OH-D_3	142 (18)	131 (5)	157 (23)	125 (6)	1.0-150	$2.68\cdot10^{-2} \pm 3.79\cdot10^{-3}$	$2.66\cdot10^{-2} \pm 1.89\cdot10^{-2}$	0.9803
1-OH-D_3	92 (1)	92 (6)	61 (9)	74 (11)	1.0-150	$1.38\cdot10^{-2} \pm 5.19\cdot10^{-4}$	$5.9910^{-5} \pm 5.68\cdot10^{-4}$	0.9952
1-OH-D_2	100 (4)	97 (2)	70 (8)	68 (8)	2.5-150	$1.95\cdot10^{-2} \pm 5.25\cdot10^{-4}$	$6.82\cdot10^{-5} \pm 5.75\cdot10^{-4}$	0.9956
24,25-$(OH)_2$-D_3	94 (4)	84 (5)	99 (13)	99 (11)	1.0-150	$6.63\cdot10^{-3} \pm 2.98\cdot10^{-4}$	$4.46\cdot10^{-4} \pm 3.26\cdot10^{-4}$	0.9928
1,25-$(OH)_2$-D_2	94 (3)	96 (3)	99 (8)	109 (8)	1.0-150	$6.90\cdot10^{-3} \pm 2.53\cdot10^{-4}$	$-2.92\cdot10^{-4} \pm 2.77\cdot10^{-4}$	0.9966
1,25-$(OH)_2$-D_3	79 (54)	83 (2)	95 (17)	111 (617%)	2.50-150	$1.06\cdot10^{-2} \pm 6.09\cdot10^{-4}$	$-5.81\cdot10^{-4} \pm 6.67\cdot10^{-4}$	0.9941

R^2: Determination coefficient. S_a and S_b: Standard deviations of slope and intercept respectively. Palmitate-D_3-$^{13}C_{16}$ was used as IS for ester metabolites and 25-OHD_3-$^{13}C_5$ for the rest of compounds. a) Results obtained following the Matuszewski method[27] as an average (n = 5) of each analyte at two concentration levels (15 and 150 µg/L). b) Concentration of the analytes in the matrix = 15 µg/L. c) Concentrations of the analytes in the matrix = 150 µg/L. Palmitate-D_3-$^{13}C_{16}$ was used as surrogate for ester metabolites and 25-OHD_3-$^{13}C_5$ for the rest of compounds.
